# Proteomic Profile of Procoagulant Extracellular Vesicles Reflects Complement System Activation and Platelet Hyperreactivity of Patients with Severe COVID-19

**DOI:** 10.3389/fcimb.2022.926352

**Published:** 2022-07-22

**Authors:** Emilly Caroline dos Santos Moraes, Remy Martins-Gonçalves, Luana Rocha da Silva, Samuel Coelho Mandacaru, Reynaldo Magalhães Melo, Isaclaudia Azevedo-Quintanilha, Jonas Perales, Fernando A. Bozza, Thiago Moreno Lopes Souza, Hugo Caire Castro-Faria-Neto, Eugenio D. Hottz, Patricia T. Bozza, Monique R. O. Trugilho

**Affiliations:** ^1^ Laboratory of Toxinology, Oswaldo Cruz Institute, FIOCRUZ, Rio de Janeiro, Brazil; ^2^ Laboratory of Immunopharmacology, Oswaldo Cruz Institute, FIOCRUZ, Rio de Janeiro, Brazil; ^3^ Center for Technological Development in Health, Oswaldo Cruz Foundation, Rio de Janeiro, Brazil; ^4^ Laboratory Protein Chemistry and Biochemistry and Laboratory of Gene Biology, Department of Cell Biology, University of Brasília, Brasília, Brazil; ^5^ National Institute of Infectious Disease Evandro Chagas, Oswaldo Cruz Foundation, Rio de Janeiro, Brazil; ^6^ D’Or Institute for Research and Education, Rio de Janeiro, Brazil; ^7^ Laboratory of Immunothrombosis, Department of Biochemistry, Federal University of Juiz de Fora, Juiz de Fora, Brazil

**Keywords:** Extracellular vesicles, LC-MS/MS, proteome, SARS-CoV-2, platelets, complement factors, tissue factor

## Abstract

**Background:**

Extracellular vesicles (EVs) are a valuable source of biomarkers and display the pathophysiological status of various diseases. In COVID-19, EVs have been explored in several studies for their ability to reflect molecular changes caused by SARS-CoV-2. Here we provide insights into the roles of EVs in pathological processes associated with the progression and severity of COVID-19.

**Methods:**

In this study, we used a label-free shotgun proteomic approach to identify and quantify alterations in EV protein abundance in severe COVID-19 patients. We isolated plasma extracellular vesicles from healthy donors and patients with severe COVID-19 by size exclusion chromatography (SEC). Then, flow cytometry was performed to assess the origin of EVs and to investigate the presence of circulating procoagulant EVs in COVID-19 patients. A total protein extraction was performed, and samples were analyzed by nLC-MS/MS in a Q-Exactive HF-X. Finally, computational analysis was applied to signify biological processes related to disease pathogenesis.

**Results:**

We report significant changes in the proteome of EVs from patients with severe COVID-19. Flow cytometry experiments indicated an increase in total circulating EVs and with tissue factor (TF) dependent procoagulant activity. Differentially expressed proteins in the disease groups were associated with complement and coagulation cascades, platelet degranulation, and acute inflammatory response.

**Conclusions:**

The proteomic data reinforce the changes in the proteome of extracellular vesicles from patients infected with SARS-CoV-2 and suggest a role for EVs in severe COVID-19.

## Introduction

As the current pandemic caused by the severe acute respiratory syndrome coronavirus 2 (SARS-CoV-2) reaches its third year, with over 540 million confirmed cases and 6 million deaths worldwide (Ritchie et al., 2020), COVID-19 is still the greatest global health challenge of the moment. Although clinical manifestations as well as epidemiologic data vary as the pandemic evolves worldwide (Levin et al., 2020; Nicolete et al., 2022), most cases are mild or asymptomatic. However, patients who develop severe COVID-19 often progress to acute respiratory distress syndrome, sepsis, coagulopathies (Vardhana and Wolchok, 2020), and metabolic perturbations (Krishnan et al., 2021). Over 40% of critically ill patients present with thrombotic complications, with high rates of deep vein thrombosis and pulmonary embolism (Klok et al., 2020), highlighting the hypercoagulable state associated with severe COVID-19 as a major concern for health systems worldwide (Asakura and Ogawa, 2021).

Considering the need to unravel different aspects of the clinical presentation and progression of the disease, efforts have been made by the scientific community to provide data on the pathophysiology of COVID-19 (Halawa et al., 2022; Ning et al., 2022). In previous studies, we and others have described the increase in platelet activation and platelet–leukocyte aggregates in critically ill COVID-19 patients (Manne et al., 2020; Middleton et al., 2020; Zaid et al., 2020; Nicolai et al., 2020; Hottz et al., 2022; Hottz, et al., 2020). We have now expanded our investigations, focusing on the role of plasma-derived EVs in COVID-19.

Extracellular vesicles (EVs) have been important targets in recent decades (Yáñez-Mó et al., 2015). EVs are particles delimited by a lipid bilayer widely distributed in circulation and act as communication signals between cells and are released by various activated cell types (Mathieu et al., 2019). These structures reflect the physiological state of the cells of origin and are regarded not only as intercellular communicators but also as key players in the development and pathophysiology of a wide variety of diseases (Shao et al., 2018).

The EV proteome has been identified and quantified in different pathological events, including cancer (M.-Y. Li et al., 2021), sepsis (Morris et al., 2020), and chronic obstructive pulmonary disease (Koba et al., 2021). Those studies point to the discovery of biomarkers and metabolic pathways that are modulated by the action of extracellular vesicles (Yáñez-Mó et al., 2015). In COVID-19, vesicles isolated from patient plasma have been associated with immune status, inflammation, and clotting, suggesting that these events remain critical and should be monitored carefully even after recovery (Mao et al., 2021). Moreover, exosome proteins were also strongly correlated with disease severity and identified as potential biomarkers, being able to discriminate between COVID-19 patients and healthy donors (Barberis et al., 2021).

Notably, these studies add evidence to the direct role of EVs in understanding the molecular mechanisms of disease. Therefore, in our work, we use a method that isolates EVs in the 50–100 nm size range, which includes not only exosomes but all plasma vesicles, as described by Böing et al. (2014), and performs a label-free shotgun proteomic approach to identify and quantify any alterations in protein abundance. By distinguishing the biological groups in severe COVID-19 patients (survivors (S) and non-survivors (NS)) and healthy volunteers (C), we provide insights into the pathological processes associated with the progression of COVID-19. Finally, we propose that proteome characterization of EVs can be used to signal proteins characteristic of severe COVID-19 and contribute to the understanding of the pathophysiology of COVID-19.

## Methods

### Study Design

This study was conducted in accordance with the Declaration of Helsinki and approved by the National Review Board of Brazil (Comissão Nacional de Ética em Pesquisa CONEP (CAAE: 30650420.4.1001.0008). After the obtaining of written consent from participants or their representatives, blood samples from severe COVID-19 patients were collected up to 72 h from ICU admission at three reference centers (Instituto Estadual do Cérebro Paulo Niemeyer, Hospital Copa Star and Leblon Campaign Hospital, all in Rio de Janeiro, Brazil), whose characteristics are presented in **Tables 1**, **2**. Severe COVID-19 was defined as critically ill patients presenting with viral pneumonia on a computed tomography scan and requiring oxygen supplementation through either a nonrebreather mask or mechanical ventilation (Akhvlediani et al., 2020). All patients had a confirmed diagnosis of SARS-CoV-2 through RT-PCR of nasal swabs and/or tracheal aspirates. The primary outcome of patients was registered after 28 days, and their demographic data, clinical data, and the necessity of invasive mechanical ventilation were retrieved from their electronic medical record. Peripheral vein blood was also collected from 18 SARS-CoV-2-negative participants as tested by RT-qPCR on the day of blood sampling.

**Table 1:** Clinical Characteristics of COVID-19 patients and control subjects in the proteome cohort.

Characteristics	Control (5)	COVID-19 (10)	COVID-19 Survivors (5)	COVID-19 Non-survivors (5)
Age (years)	43 (35.5–63.5)	50 (45.2–62.5)	47 (39.5–62.5)	53 (48–65.5)
Sex (male)	2 (40%)	4 (40%)	2 (40%)	2 (40%)
SAPS 3	–	60.5 (55.75–80.25)	56 (42–59)	80 (60.5–82.5)#
PaO2/FiO2 (ratio)	–		172 (164–521.9)	135.4 (86.95–144.5)
Time from symptom onset to blood collection	–	13 (10–18)	15.5 (8.75–21.5)	12 (10–16)
**RESPIRATORY SUPPORT**				
Mechanical Ventilation	–	9 (90%)	4 (80%)	5 (100%)
Oxygen Supplementation	–	1 (10%)	1 (20%)	0 (0%)
**COMORBITIES**				
Heat Disease	0 (0%)		0 (0%)	0 (0%)
Diabetes	0 (0%)	3 (30%)	2 (40%)	1 (20%)
Cancer	0 (0%)	3 (30%)	2 (40%)	1 (20%)
Obesity	1 (20%)	2 (20%)	1 (20%)	1 (20%)
**PRESENTING SYMPTOMS**				
Cough	–	10 (100%)	5 (100%)	5 (100%)
Fever	–	10 (100%)	5 (100%)	5 (100%)
Dyspnea	–	10 (100%)	5 (100%)	5 (100%)
Headache	–	4 (40%)	3 (60%)	1 (20%)
Anosmia	–	6 (60%)	3 (60%)	3 (60%)
**LABORATORY FINDINGS ON ADMISSION**				
Leukocytes (cells/mm^3^)	–	16,070 (12,075–27,290)	17,140 (12,028–24,360)	16,070 (10,870–33,100)
Lymphocytes (cells/mm^3^)	–	1,286 (637–1,527)	1,215 (882–1,555)	1,286 (211–1,662)
Monocytes (cells/mm^3^)	–	699 (525–860)	687 (450–894)	712 (556–1,170)
Platelets (10^3^/mm^3^)	–	265 (173–336.5)	336.5 (286–578.3)	193 (117–239.5)#
C-reactive protein (mg/L)	0.13 (0.1–0.19)	16.45 (11.53–25.32)**	14.41 (8.11–21.09)	20.33 (14.96–25.59)
Fibrinogen (mg/dl)	287 (281–299)	563.5 (500.6–632.6)**	581 (528–643)	545 (528–643)
D-dimer (ng/ml)	456 (328–994)	5,220 (3,083–19,011)**	3,464 (1,991–5220)	4,929 (16,316–31,048) #
IL-6 (pg/ml)	7 (0.1–11)	42 (23.5–69.25)*	24 (16–62)	56 (34–160.5)

Quantitative variables are represented as the median and interquartile range, and qualitative variables as absolute number and percentage. Qualitative variables were compared using the 2-tailed Fisher exact test, and the numerical variables using the Student’s t-test for parametric and the Mann–Whitney U test for nonparametric distributions. Statistical differences between COVID-19 patients and healthy donors are indicated as *p <0.05 and **p <0.01. Statistical differences between COVID-19 survivors and COVID-19 non-survivors are indicated as ^#^p <0.05.

**Table 2:** Clinical Characteristics of COVID-19 patients and control subjects in the flow cytometry cohort.

Characteristics	Control (14)	COVID-19 (18)	COVID-19Survivors (9)	COVID-19Non-survivors (9)
Age (years)	56 (44–61)	63 (52–77)	64 (49.5–80)	62 (54–78)
Sex (male)	7 (54%)	11 (61%)	6 (66%)	5 (55%)
SAPS 3	–	60 (47–71.75)	47 (43–60)	73 (64–76)#
PaO2/FiO2 (ratio)	–	138.8 (98–164)	121.1 (95.57–158.3)	138 (98–206.3)
Time from symptom onset to blood collection	–	10 (7.5–15)	10 (7.5–13.5)	10 (7–17)
**RESPIRATORY SUPPORT**				
Mechanical Ventilation	–	12 (66%)	4 (45%)	8 (88%)
Oxygen Supplementation	–	6 (33%)	5 (55%)	1 (11%)
**COMORBIDITIES**				
Heat Disease	0 (0%)	1 (5.5%)	0 (0%)	1 (11%)
Diabetes	0 (0%)	2 (11%)	0 (0%)	2 (22%)
Cancer	0 (0%)	0 (0%)	0 (0%)	0 (0%)
Obesity	0 (0%)	2 (11%)	1 (11%)	1 (11%)
**PRESENTING SYMPTOMS**				
Cough	–	14 (77%)	7 (77%)	7 (77%)
Fever	–	14 (77%)	7 (77%)	7 (77%)
Dyspnea	–	14 (77%)	7 (77%)	7 (77%)
Headache	–	5 (27%)	2 (22%)	3 (33%)
Anosmia	–	9 (50%)	4 (45%)	5 (55%)
**LABORATORY FINDINGS ON ADMISSION**				
Leukocytes (cells/mm^3^)	–	11,500 (1,991–20,850)	11,600 (9,425–16,925)	12,395 (1,939–22,450)
Lymphocytes (cells/mm^3^)	–	990.5 (317.3–1,514)	801 (146.3–1,364)	1,035 (317.3–2,305)
Monocytes (cells/mm^3^)	–	821.5 (550.8–996.3)	608 (373–881)	854 (655–1,208)
Platelets (10^3^/mm^3^)	–	184 (137–229.5)	195.5 (164.5–319.5)	174.5 (101.8–228.5)
C-reactive protein (mg/L)	0.11 (0.1–0.19)	18.2 (3.4–26.6)**	6.4 (1.8–18.4)	25 (12.2–29.9)
Fibrinogen (mg/dl)	281 (236–287)	440.5 (375.3–545.3)*	375.3 (232.7–466.6)	512 (401.6–583.4)
D-dimer (ng/ml)	257 (241–994)	3,090 (1,811–14,732)**	1,662 (511–2499)	7,401 (3,090–26,033)#
IL-6 (pg/ml)	13 (7–17)	34.5 (16.25–123.3)	20 (5.5–80)	70 (32–127.5)

Quantitative variables are represented as the median and interquartile range, and qualitative variables as absolute number and percentage. Qualitative variables were compared using the 2-tailed Fisher exact test, and the numerical variables using the Student’s t-test for parametric and the Mann–Whitney U test for nonparametric distributions. Statistical differences between COVID-19 patients and healthy donors are indicated as *p <0.05 and **p <0.01. Statistical differences between COVID-19 survivors and COVID-19 non-survivors are indicated as ^#^p <0.05.

### Blood Sample Collection and Plasma Obtention

Subjects had 17 ml of blood collected into syringes containing 3 ml of Acid-Citrate-Dextrose (ACD). Blood samples were centrifuged at 150×*g* for 20 min at 25°C. The supernatant (platelet-rich plasma—PRP) was supplemented with 100 nM prostaglandin E1 (PGE1, Cayman 13010) and centrifuged at 500×*g* for 20 min at 25°C to obtain platelet-poor plasma (PPP). Platelet depleted plasma was obtained through centrifugation of the PPP at 2,500×*g* for 20 min. Plasma samples were stored at −80°C.

### Extracellular Vesicle Isolation Through Size Exclusion Chromatography (SEC)

One milliliter of platelet depleted plasma samples were loaded into a PD-10 column, filled with 10 ml of Sepharose CL-2B (Sigma Aldrich) and prewashed with 50 ml of sterile PBS for size exclusion chromatography separation. Each sample was loaded individually into columns and PBS was used to elute the fractions, collected in microtubes with 500 μl of effluent from each one. Enriched EV fractions (6 to 11) were pooled into a tube and stored at −20°C, according to Böing et al. (2014).

### Extracellular Vesicles Isolation Through Serial Centrifugation

EVs were obtained trhough differential centrifugation according to the international guidelines for EVs (Coumans et al., 2017; Théry et al., 2018; Mendes-de-Almeida et al., 2021). Briefly, 1 ml of platelet-depleted plasma samples from healthy donors or COVID-19 survivors were slowly thawed and centrifuged at 16,100×*g* at 4°C for 60 min. The top 950 µl of the supernatant was removed, and 950 µl of 0.22 µM filtered DPBS was added. The samples were centrifuged again (same parameters), the top 950 µl of the supernatant was removed, and the remaining 50 ul containing the EVs were stored at −80°C.

### Extracellular Vesicle Characterization

EV samples were slowly thawed in ice before labeling with AnnexinV-FITC (347523) or AnnexinV-450 (560506) for EV identification; antibodies anti-CD45-BV421 (304032), CD146-PerCP (342014), CD235-APC (306608), and CD41-APC-Cy7 (303716) for the tissue of origin identification; and CD142-PE (550312) for TF expression identification. Samples were analyzed using a CytoFlex (Beckman Coulter) flow cytometer, and EVs were gated using 1 µm polystyrene beads in a violet side scatter detector setup. Isotype-matched immunoglobulins and anti-CD41 antibodies conjugated with all fluorochromes were used for negative controls and correct color compensation, respectively, and data analysis was performed using the CytExpert software (Beckman Coulter).

### Statistical Analysis

The distribution of different variables was assessed using the Shapiro–Wilk test. Comparisons between groups were performed using the Student t-test for parametric distributions and the Mann–Whitney U test for nonparametric distributions. All statistical analyses were performed using GraphPad Prism version 8.1. Variables are represented as the medians, with whiskers indicating the 25th and 75th quartiles. Statistical significance is represented as *p <0.05 and **p <0.001.

### Shotgun Proteomics

#### Sample Preparation for Mass Spectrometry

Samples were prepared as previously described by De-Azambuja-Rodrigues et al. (2021). EVs were suspended in RapiGest SF^®^ (Waters) at 0.1% (w/v) in 50 mM ammonium bicarbonate. Aliquots of 50 μg of protein were reduced in dithiothreitol (3 h at 37°C, 10 mM final concentration), alkylated in iodoacetamide (30 min in the dark at room temperature, 25 mM final concentration), and digested with Trypsin (Promega, USA) (overnight at 37°C, 1:50 (m/m). The tryptic peptides were desalted with POROS R2 resin (Applied Biosystems), eluted in TFA 0.1% containing acetonitrile 70% (v/v) and dried in a vacuum centrifuge. The purified peptides were resuspended in a 1% (v/v) formic acid (FA) solution and stored at −20°C until mass spectrometric analysis.

#### Mass Spectrometry Analysis

The nLC-nESI MS/MS analysis was performed on an Ultimate 3000 (Dionex) chromatographic system coupled to a Q-Exactive HF-X mass spectrometer (Thermo). About 1 μg of the tryptic digest was applied to a guard column (2 cm × 100 μm internal diameter × 3 μm particle size Magic C18 AQ, Michrom Bioresources) followed by an analytical column (25.5 cm PicoFrit™ Self-Pack, New Objective × 75 μm internal diameter × 1.9 μm particle size ReproSil-Pur 120 C18-AQ, DR. MAISCH). Mobile phase A (0.1% v/v formic acid in water) and mobile phase B (0.1% v/v formic acid in acetonitrile) were used in a separation gradient from 2 to 40% B for 158 min; concentration was increased to 80% B in 4 min and maintained isocratically for 2 additional minutes. The spray voltage was adjusted to 1.9 kV in the nanoelectrospray source with no auxiliary gas flow and the capillary temperature set to 250°C. The lens voltage was set to 60 V. MS1 spectra were acquired in the profile mode in the Orbitrap analyzer (m/z 300 to 1,500) with a resolution of 70,000 FWHM (Full-Width Half Maximum, m/z 200) and Automatic Gain Control (AGC) set to 1 × 10^6^ and maximum injection time of 250 ms. Up to 12 precursor ions per MS1 spectrum were selected for fragmentation with higher-energy collisional dissociation (HCD) with a normalized collision energy (NCE) of 35 and an activation time of 50 ms. The isolation window was set to 2 m/z and the dynamic exclusion configured to 60 s. MS2 spectra were acquired in the Orbitrap at a resolution of 17,500 FWHM; AGC was set to 5 **×** 10^4^, maximum fill of 50 ms, intensity threshold of 1 **×** 10^5^ counts. Singly charged and unassigned ions were not subjected to fragmentation. Data were obtained in technical triplicate using Xcalibur software (version 4.2.47). The mass spectrometer was externally calibrated using a mixture composed of caffeine, MRFA peptide, and Ultramark 1621, as recommended by the instrument manufacturer. The mass spectrometry data have been deposited in the ProteomeXchange Consortium *via* the PRIDE (Perez-Riverol et al., 2019) partner repository under the identifier PXD032808.

#### Label-Free Protein Identification and Quantification

Quality control and statistical analysis were carried out individually for these comparisons. Quantification was performed using Extracted-Ion Chromatogram (XIC) through the quantification module in PEAKS X Pro (Bioinformatics Solutions Inc., Ontario, Canada). Changes observed among LC-MS/MS runs were compared according to different conditions. This analysis was set at 10 ppm for peptide mass tolerance and 0.02 Da for fragment mass tolerance. Trypsin as the enzyme with two missed cleavages allowed; cysteine carbamidomethylation as a fixed modification, and oxidation of methionine as a variable modification. High confidence peptides were used for protein identification by setting a target false discovery rate (FDR) threshold of 1% at the peptide level. Proteins that had at least one unique peptide were used for protein identification. The database was downloaded from UniProt (Reviewed Swiss-Prot) and contains 20,375 entries, available at https://www.uniprot.org/uniprot/.

#### Statistical Analysis

Peptide abundances were exported from PEAKS after the identification filters. The resulting datasets were imported to the R environment, assisted by the R studio, and analyzed using the pmartR package (Webb-Robertson et al., 2010) for quality control and statistical analysis. First, data were log2 transformed and peptides were filtered. Peptide filters were based on the presence of degenerated peptides, the presence of missing values (NA), and susceptibility to ANOVA and *g*-test (Stratton et al., 2019). Filters resulted in the removal of peptides identified as more than one protein, peptides with three or more NA values, and peptides unsusceptible to ANOVA or *g*-test. After filtering, individuals were subjectedto outlier robust Mahalanobis distance (RMD) analysis, based on median absolute deviation (MAD), skewness, correlation, and the proportion of missing values. Then, abundance data were normalized according to the SPANS tool (Webb-Robertson et al., 2011) present in pmartR. The highest score subset and normalization strategy was chosen. In the case of draws, the approach with a greater number of peptides was selected. Principal component analysis (PCA) and correlation plots were performed to visualize differences between groups and replicate reproducibility. Protein rollup was conducted with the Rrollup tool (Polpitiya et al., 2008) in pmartR with default parameters. Comparisons of biological groups were subjected to independence of missing data and ANOVA (IMD-ANOVA) statistical tests or by cluster analysis using VSClust (Schwämmle and Jensen, 2018). Networks were displayed by Cytoscape software (version 3.3.1) (Otasek et al., 2019).

## Results

### Subjects’ Characterization

Between the 15th of March and the 15th of December 2020, blood samples were collected from 27 severe COVID-19 patients and 18 control subjects. Different sets of samples were used for proteomics (**Table 1**) and flow cytometry (**Table 2**) assays.

For proteomic analysis of EVs, control subjects (5) had a median age of 43 (IQR, 35.5–63.3) years, ranging from 35 to 75 years of age. COVID-19 survivors (5) had a median of 47 (IQR, 39.5–62.5) years, ranging from 39 to 67 years, and non-survivors (5) had a median of 53 (IQR, 48–65.5) years, ranging from 48 to 70 years. All non-survivors and 80% (4) of the survivors required invasive mechanical ventilation, while a survivor required oxygen supplementation with a non-rebreather mask. Flow cytometric analysis of EVs included samples from 13 controls and 18 COVID-19 patients, including 9 survivors and 9 non-survivors. Seven (54%) of the control subjects were men, with a median age of 56 (IQR, 44–61) years, ranging from 34 to 77 years. Six (66%) of the COVID-19 survivors were male, with a median age of 64 years (IQR, 49.5–80), ranging from 38 to 93 years of age, while 5 (55%) of the COVID-19 non-survivors were male and had a median age of 62 (54–78) years of age, ranging from 51 to 81 years. For both cohorts, no significant differences were found in age, sex distribution, and comorbidities.

In accordance with other cohorts of hospitalized severe COVID-19 patients (Tang et al., 2020; Hottz et al., 2020; Malik et al., 2021), both cohorts used in this study presented elevated circulating levels of C-reactive protein (CRP), fibrinogen, and D-dimer (**Tables 1**, **2**). Additionally, COVID-19 non-survivors also presented higher levels of D-dimer on the date of hospital admission compared with patients who survived.

### Increased Plasma Levels of TF and TF-Bearing EVs in COVID-19

**Figure 1:**
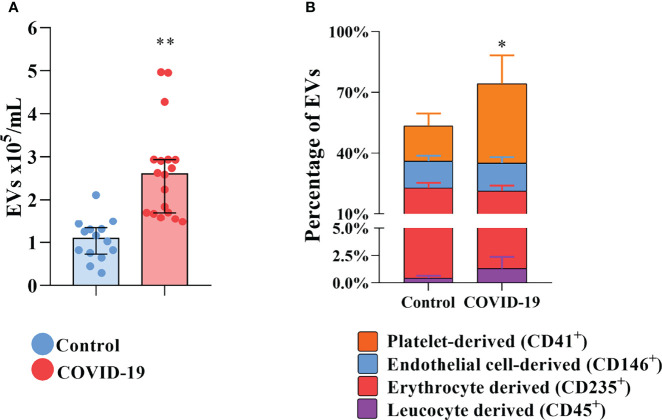
Increased circulation of EVs in COVID-19 patients. **(A)** The total concentration of EVs found in the plasma of healthy donors and COVID-19 patients. **(B)** The distribution of the circulating EVs into its tissue of derivation. * indicates the difference between the percentage of platelet-derived EVs percentage when compared to healthy donors. Medians with interquartile range are shown. Individual values for healthy donors (blue dots) and COVID-19 patients (red dots) are shown. *p<0.05 and **p<0.001.

To shed light on the presence of circulating procoagulant EVs in COVID-19 patients and their origin, EVs were isolated and labeled for flow cytometry for surface markers of their cells of origin. In comparison with control subjects, the plasma exhibits elevated concentrations of total circulating EVs of COVID-19 patients (**Figure 1A**). The plasmatic concentrations of EVs derived from platelets, endothelial cells, erythrocytes, and leukocytes are all elevated in COVID-19 patients compared with healthy donors (data not shown). However, only platelet-derived EVs were significantly increased in percentage (**Figure 1B**), becoming more representative during severe COVID-19.

**Figure 2:**
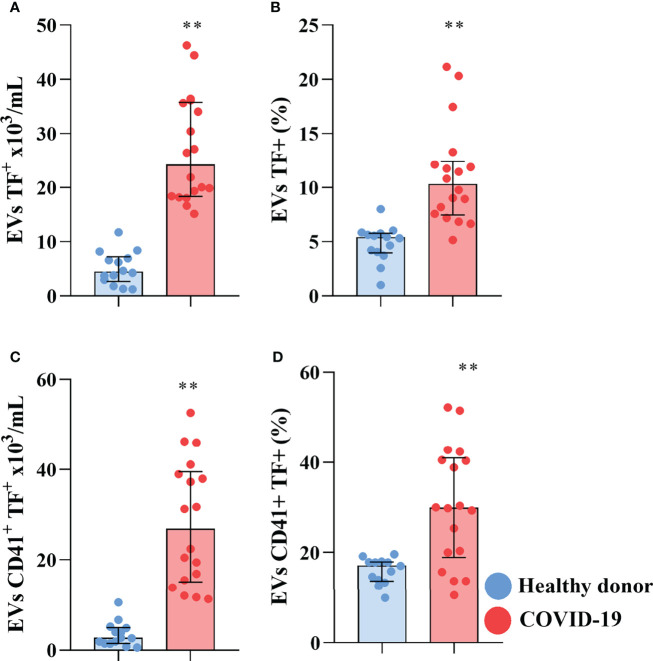
Severe COVID-19 patients present increased circulation of pro-coagulant TF-bearing EVs. The concentration **(A)** and percentage of EVs expressing TF **(B)**. The concentration **(C)** and percentage of platelet derived EVs expressing TF **(D)**. Medians with interquartile range are shown. Individual values for healthy donors (blue dots) and COVID-19 patients (red dots) are shown. **p<0.001.

Other than their luminal cargo, EVs are also capable of signaling through molecules expressed on their surface. Increased plasmatic levels of EVs, and especially of pro-coagulant tissue factor (TF) bearing EVs, are characteristic of various cancers and have been shown to be involved in metastasis and the development of DVT in cancer patients (Tesselaar et al., 2007; Tesselaar et al., 2009; Manly et al., 2010). Moreover, increased expression of TF in circulating EVs has been shown to correlate with mortality in patients infected with HIV (Mayne et al., 2012), influenza A/H1N1 (Rondina et al., 2016), and COVID-19 (Rosell et al., 2021). We investigated the presence of TF-bearing EVs in the plasma of severe COVID-19 patients and found that not only was there an increased concentration of TF^+^ EVs in the plasma of the patients (**Figure 2A**), but also that TF-bearing EVs increased their representativity, with an increased percentage of circulating EVs expressing TF on their surface compared to control (**Figure 2B**). Interestingly, of the tissues of origin investigated, only platelet-derived TF-bearing EVs presented an increase in concentration and percentage in the plasma of the COVID-19 patient (**Figures 2C, D**), indicating that platelet-derived EVs circulate with abnormal expression of TF, an important activator of the coagulation cascade. However, we could not detect the tissue of origin of all circulating EVs, including those that present reduced representability in COVID-19 patients, indicating that other tissues may also contribute to the circulating EV population.

Although anucleated, platelets carry TF pre-mRNA, which upon activation can be spliced into mRNA, releasing EVs with TF-dependent procoagulant activity (Rondina et al., 2016). Increased platelet activation has been observed in severe COVID-19 patients (Hottz et al., 2020; Manne et al., 2020; Hottz et al., 2022), which could lead to increased release of EVs expressing transmembranar TF.

**Figure 3:**
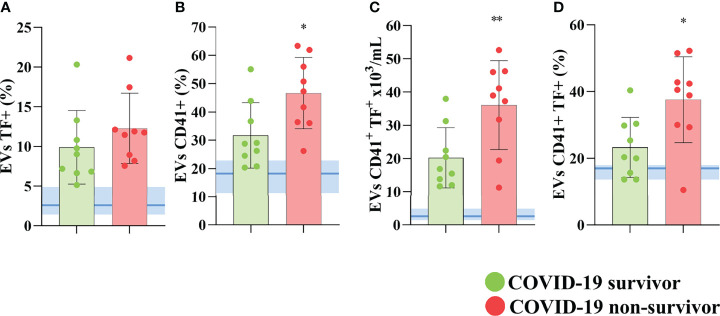
TF-bearing platelet-derived EVs correlates with poor outcome in COVID-19 patients. The percentage of total circulating **(A)** and platelet derived EVs **(B)** of COVID-19 survivors and non-survivors. The concentration of TF-bearing platelet-derived EVs **(C)** and the percentage of platelet-derived EVs expressing TF **(D)** are shown for covid survivors and non-survivors. Medians with interquartile range are shown. Individual values for COVID-19 survivors (green dots), and COVID-19 non-survivors (red dots) are shown. The blue line and shade indicate the median and interquartile range of the healthy donor. *p<0.05 and **p<0.001.

Further, we observed that although elevated in COVID-19 patients, the percentage of total circulating EVs (data not shown) and EVs expressing TF (**Figure 3A**) was not statistically different between non-survivors and survivors. However, the levels of platelet-derived EVs in the plasma of these patients (**Figure 3B**), the concentration (**Figure 3C**), and the percentage (**Figure 3D**) of platelet-derived TF-bearing EVs were significantly increased in those patients who did not survive SARS-CoV-2 infection, indicating that this population of circulating EVs may not only become more abundant during the disease development but also that it may play an important part in the severity and mortality in COVID-19 patients.

### Proteomic Profile of EVs in COVID-19

**Figure 4:**
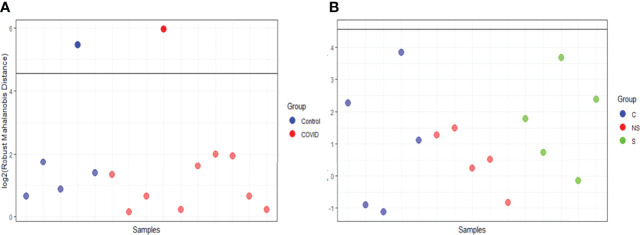
Robust Mahalanobis distance analysis. The threshold of 1e−04, represented by the line in the graph, corresponds to outliers. **(A)** RMD of Control vs COVID. **(B)** RMD of C vs S vs NS. Images analysis and creation performed in pmartR. The Control group (C) is represented by blue dots, COVID-19 survivors (S) by green dots, and COVID-19 non-survivors (NS) by red dots.

EV was isolated from 15 samples and classified as the following: 5 controls from healthy donors (C), 5 from COVID-19 patients that survived (S), and 5 from patients that did not survive COVID-19 (NS). Three comparisons were assessed: C vs COVID-19; S vs NS; and C vs S vs NS. We used the robust Mahalanobis distance analysis to assess outliers between the samples for two sets of comparisons between the groups: C vs COVID-19 and C vs S vs NS. The result of the analysis suggests that 1 control sample (C4) and 1 sample from the survivor group (S3) are extreme outliers in the comparison of C vs COVID-19 (**Figure 4A**). Even so, no sample was considered an outlier in the comparison of C vs S vs NS (**Figure 4B**). For this reason, we chose to keep the entire set of samples in this and the next analyses.

**Figure 5:**
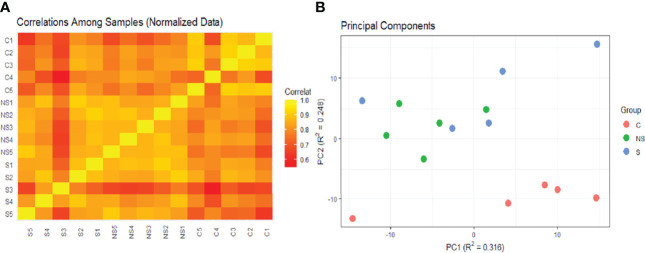
Heatmap of correlations and PCA of samples. **(A)** Heatmap constructed based on Pearson correlation matrix. **(B)** Probabilistic PCA. Both images were analyzed and created in pmartR. The Control group (C) is represented by red dots, COVID-19 survivors (S) by blue dots, and COVID-19 non-survivors (NS) by green dots.

Correlation analysis and PCA suggested the low correlation of these two samples with other groups (**Figure 5**). The correlation heatmap showed greater correlation of control group replicates with each other than with COVID-19 group replicates (**Figure 5A**). Moreover, principal component analysis of EV protein abundance demonstrated that the C was well segregated from the S and NS (**Figure 5B**).

**Figure 6:**
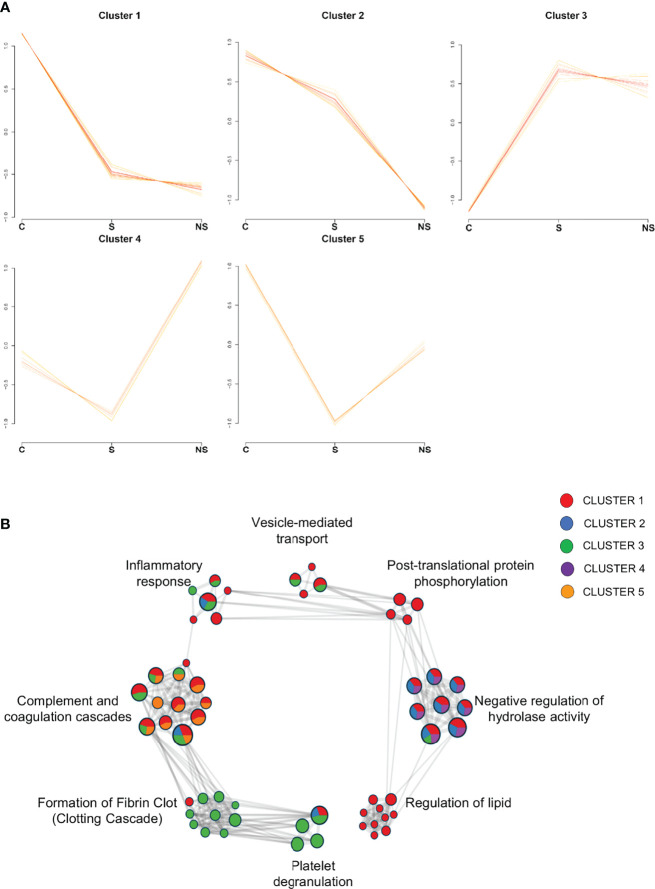
Clusters formed by C vs S vs NS comparison and Network of enriched pathways. **(A)** Variance sensitive clustering analysis performed in VSClust, which defined the optimum number of clusters as five. **(B)** Network of enriched terms from protein lists of clusters colored by cluster ID, where nodes that share the same ID are typically close to each other. Pathways database used in Metascape: WikiPathways, GO Biological Process, KEGG, and Reactome. Control group (C), COVID-19 survivors (S), and COVID-19 non-survivors (NS).

Initially, to explore the general EVs proteome changes, 220 proteins were confidently quantified in the C vs S vs NS comparison. The cluster analysis resulted in the formation of five clusters, each built of 66 proteins with different abundance profiles across conditions (**Figure 6**). Twenty-one clustered proteins from patients had a pattern of downregulation in COVID-19 compared to controls, while proteins from cluster 3 showed a different pattern, with proteins upregulated in survivors and non-survivors. Interestingly, in cluster 2, 16 proteins showed a pattern of downregulation as the disease progressed (control → survivor → non-survivor). A list containing all information regarding protein clusterization can be found in **Supplemental Table 1**.

To understand the biological processes involved in EV functions regarding the clustered proteins, we performed over representation analysis (ORA) analyses in Metascape, using KEGG, Reactome, WikiPathways, and GO Biological Process databases. Enriched pathway terms of the clusters were associated with the complement and coagulation cascade, platelet degranulation, regulation of lipid, negative regulation of hydrolases activity, post-translational protein phosphorylation, vesicle-mediated transport, formation of fibrin clot, and inflammatory response (**Figure 6B**). Additionally, the distribution of proteins from cluster IDs can be observed among pathway terms, in which platelet degranulation and clotting cascade are associated with cluster 3, with proteins upregulated in S and NS, while regulation of lipid and post-translational protein phosphorylation is associated with proteins from cluster 1, specifically, in which set of proteins is downregulated in clinical conditions.

### The EV Proteome is Altered During SARS-CoV-2 Infection

**Figure 7:**
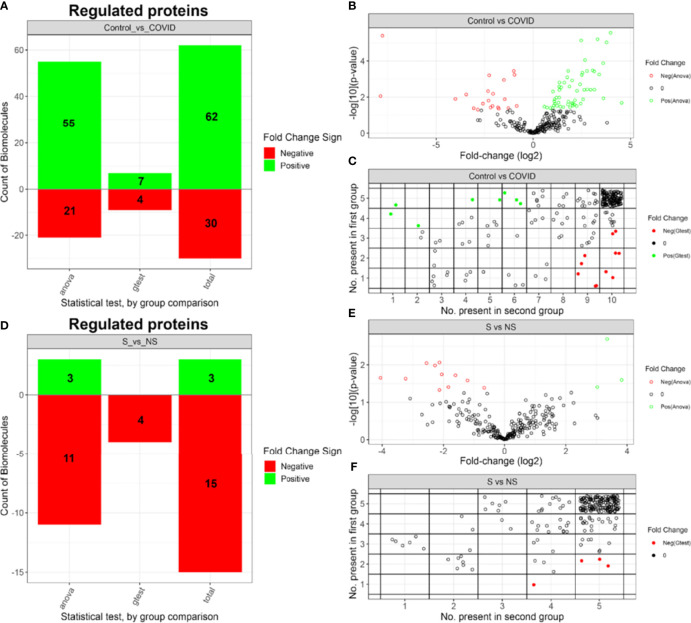
Summary of IMD-ANOVA regulated proteins. **(A)** Number and FC signal of regulated proteins identified by each approach in C vs COVID-19 comparison. **(B)** Volcano plot of C vs COVID-19 comparison. **(C)** Number of proteins observed in each control and COVID-19 groups. **(D)** Number and FC signal of regulated proteins identified by each approach in S vs NS groups. **(E)** Volcano plot of S vs NS comparison. **(F)** Number of proteins observed in each S vs NS groups. Control group (C), COVID-19 survivors (S), and COVID-19 non-survivors (NS).

After proteome label-free quantification, proteins were subjected to IMD-ANOVA analysis to highlight the differentially expressed proteins. A set of 92 differentially expressed proteins (DEPs) were regulated in the C vs COVID comparison (**Figure 7A**), with 62 proteins upregulated in the control group and 30 proteins upregulated in the COVID-19 group. Moreover, 44 of the DEPs presented a fold change (FC) greater than 2 (**Figure 7B**). Meanwhile, 18 proteins were regulated in the S vs NS comparison (**Figure 7D**), where 11 presented FC greater than 2 (**Figure 7E**). IMD regulated proteins can be evaluated by the number of observations in each group (**Figures 7C, F**). In this comparison, 3 proteins were upregulated in the S group while 15 proteins were upregulated in the NS group. All datasets, including fold-change for both comparisons, are provided in **Supplemental Table 2**.

**Figure 8:**
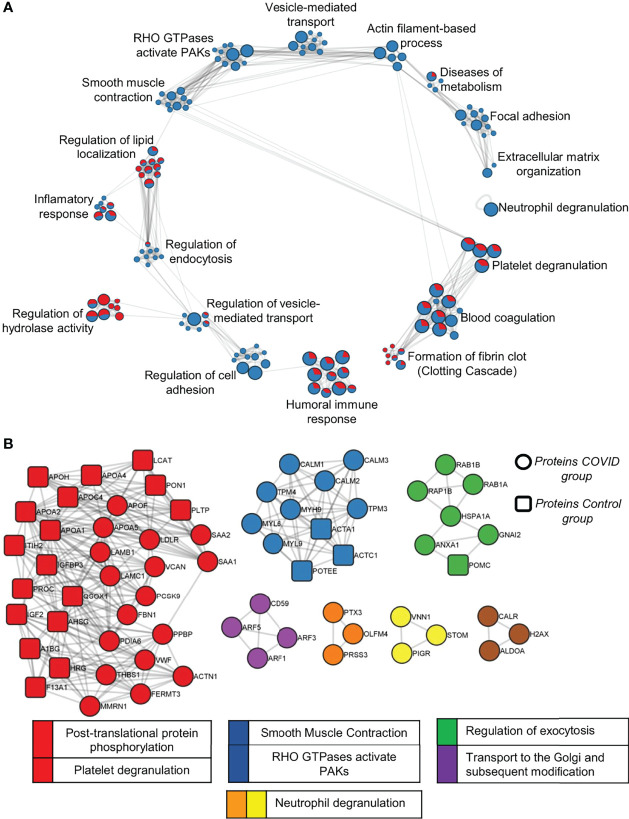
Pathway analysis of C *vs* COVID-19 proteome. **(A)** A network of enriched pathway terms from protein lists of DEPs and EPs in the C *vs* COVID-19 comparison. Nodes that share the same ID are typically close to each other. Pathways database used was WikiPathways, GO Biological Process, KEGG, and Reactome. Red, C; Blue, COVID-12. **(B)** Protein–protein interaction analysis of DEPs and EPs was performed with STRING, BioGrid, OmniPath, and InWeb_IM databases. Only physical interactions in STRING (physical score >0.132) and BioGrid are used.

The list of DEPs and exclusive proteins (EPs) of each condition in the C vs COVID-19 comparison, totaling 282 proteins in COVID-19 and 90 proteins in C, was used to perform over-representation analysis (ORA) with human pathway databases. Enriched terms were resumed in: neutrophil degranulation; platelet degranulation; blood coagulation; formation of fibrin clots (clotting cascade); humoral immune response; regulation of cell adhesion; regulation of vesicle-mediated transport; regulation of hydrolase activity; regulation of endocytosis; inflammatory response; regulation of lipid localization; smooth muscle contraction; RHO GTPases activate PAKs; vesicle-mediated transport; actin filament-based process; diseases of metabolism; focal adhesion and extracellular matrix organization (**Figure 8A**). The representation of DEPs in the EV proteome from the C and COVID-19 groups is proportionally indicated by the chart graph, with red indicating the C representation and blue indicating the COVID-19 representation. Proteins like Platelet Glycoprotein Ib—GPIBB (P13224), von Willebrand Factor—vWF (P04275), glycoproteins CD81 (P60033) and CD9 (P21926), and coagulation factor XIII (P00451), are some interesting examples of proteins that enrich terms associated with hemostasis process such as platelet degranulation; blood coagulation; formation of fibrin clot. The terms focal adhesion; extracellular matrix organization; neutrophil degranulation; regulation of cell adhesion; smooth muscle contraction; RHO GTPases activate PAKs; vesicle-mediated transport and actin filament-based process is enriched exclusively by proteins of the COVID-19 proteome, while regulation of hydrolase activity and regulation of lipid localization are enriched majority by proteins upregulated on the C group.

Additionally, protein–protein interaction (PPI) enrichment analysis was performed using the same differential protein lists for each condition to visualize subsets of proteins and their associated pathways. Circular nodes indicate proteins that belong to COVID-19, which composes most proteins among all subsets, and rectangular nodes indicate proteins that belong to the C group. Densely connected networks can be detected by Molecular Complex Detection (MCODE). Then pathway terms are applied to each MCODE component, and the best-scoring is determined as the functional description of the corresponding subset indicated by colors. Protein subset of MCODE 1, indicated by red containing CXCL7 (P02775), thrombospondin (P35442), and vWF (P04275) as examples of proteins from the proteome of the patients in this subset, associated with platelet degranulation activity and post-translational protein phosphorylation terms; MCODE 2, indicated by blue, is associated with smooth muscle contraction and RHO GTPases activate PAK terms; MCODE 3, indicated by green, is associated with the regulation of exocytosis; MCODE 4, indicated by purple, is associated with transport to the Golgi and subsequent modification terms; MCODE 5 and 6, indicated by orange and yellow, respectively, is also associated with neutrophil degranulation terms (**Figure 8B**).

### Regulation of Cell Adhesion, Neutrophil Degranulation, and Immune System are Strongly Associated With the Proteome of NS Group

**Figure 9:**
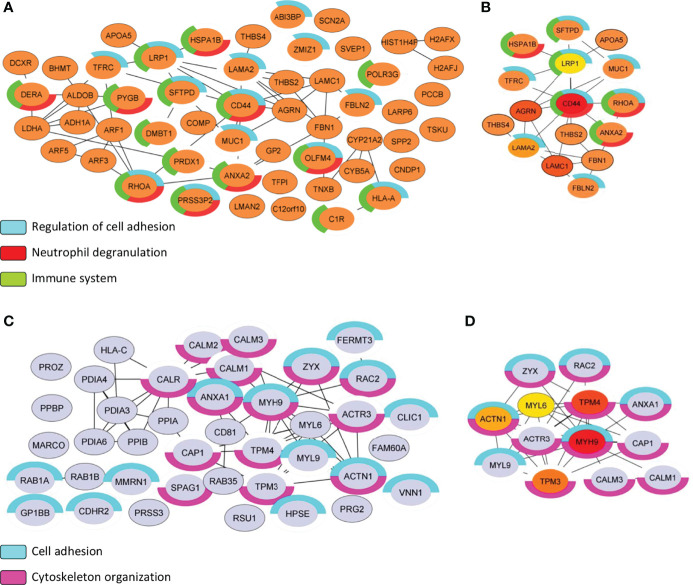
Subnetwork analysis of S vs NS EVs proteome. **(A, C)** Protein–protein interaction analysis was performed with the STRING database on Cytoscape program. Only physical interactions in STRING (physical score >0.132) were considered. For each protein list (S vs NS), functional analysis with human genome reference was applied, and terms associated with the immune system biological process were highlighted by color charts. **(B, D)** Maximal Clique Centrality (MCC) is used to discover featured nodes in the subnetwork, used through CytoHubba application. The analysis returned the top 5 central nodes of NS proteome: CD44 antigen (CD44), Agrin (AGRN), Lamin subunit gamma-1 (LAMC1), Lamin subunit gamma-2 (LAMA2), and Prolow-density lipoprotein receptor-related protein 1 (LRP1). While the top 5 central nodes of S proteome were: Myosin-9 (MYH9), Tropomyosin alpha-4 chain (TPM4), Tropomyosin alpha-3 chain (TPM3), Alpha-actinin-1 (ACTN1), and Myosin light polypeptide 6 (MYL6). Control group (C), COVID-19 survivors (S), and COVID-19 non-survivors (NS).

The protein list of differential proteomes (DEPs + EPs) from S and NS EVs totaled 85 proteins representing the proteome of the S EVs and 132 proteins representing the proteome of the NS EVs. From there, we focused on PPI analysis using String and CytoHubba applications on Cytoscape, to highlight the relevant proteins identified in each proteome (**Figure 9**). The biological process of the NS proteome shows that proteins involved in the regulation of cell adhesion, neutrophil degranulation, and the immune system (**Figure 9A**). Additionally, MCC algorithms analysis indicated that the CD44 antigen (CD44) is a central protein of the subnetwork from the NS proteome (**Figure 9B**). Similarly, the biological process of S proteome also shows proteins associated with cell adhesion and has 14 proteins associated with the cytoskeleton organization process (**Figure 9C**). MCC algorithms analysis indicates myosin-9 (MYH9) as the central protein of the current subnetwork (**Figure 9D**).

## Discussion

Previous studies have considered the potential of EVs as biomarkers and strong reflectors of pathological conditions, and recent studies have already demonstrated that EVs can be used to evaluate patients with COVID-19 (Mao et al., 2021; Barberis et al., 2021; Lam et al., 2021). To provide insights into the mechanisms associated with EVs in SARS-CoV-2 infection, we characterized the plasma EVs by flow cytometry and explored the proteomic differences between individuals with severe COVID-19 and healthy volunteers.

Here, we collected enriched fractions of circulating EVs from the plasma of severe COVID-19 and healthy volunteers. Considering that there is no gold standard method for the isolation of the vesicles, the choice of methodology is based on the objective of the study (Konoshenko et al., 2018; J. Li et al., 2019). For shotgun proteomics analyses, contamination by major plasma proteins and the minimum number of EV proteins required for mass spectrometry are critical factors (Lötvall et al., 2014; Sódar et al., 2017). For this reason, we used an SEC-based protocol that, in addition to minimizing contamination by plasma proteins (Wang et al., 2017), maintains the integrity of EVs (Stranska et al., 2018), and applies to small volumes of plasma, characteristic of experiments with clinical samples.

Then, we used an expanded cohort to assess the profile of enriched EVs in our study and found that plasma from COVID-19 patients exhibits higher concentrations of circulating EVs and TF-bearing EVs. Indeed, the increased release of EVs has already been described in SARS-CoV-2 (Balbi et al., 2021) and other infections (Fleming et al., 2014; Han et al., 2019; Pocsfalvi et al., 2020) and, furthermore, the content of proteins transported by exosomes can be altered, resulting in modulation of the immune response of the host (Yoshikawa et al., 2019). In COVID-19, most circulating EVs are derived from activated platelets, and therefore, their pro-coagulant activity has effects on the inflammatory process (Puhm et al., 2022). EVs stimulate the release of pro-inflammatory cytokines, causing endothelial damage (Lindemann et al., 2001; Puhm et al., 2021). In this context, EVs may increase platelet adhesion to collagen matrices with subsequent monocyte activation and TF production.

Tissue factor (TF), also known as coagulation factor III, is the main initiator of the extrinsic pathway of the coagulation cascade (van der Poll, 2008). TF acts as the receptor and cofactor for coagulation factor VII. After dimerization, the FIIIa/FVIIa complex can activate the coagulation factor X, leading to thrombin generation and fibrin clot formation (Grover and Mackman, 2018). TF is a transmembrane protein and can be found on the surface of a wide variety of extravascular tissues (Grover and Mackman, 2020). However, TF can also be found on the surface of EVs (Date et al., 2017). Increased presence of TF-bearing EVs has been found in the occluded coronary artery of patients with myocardial infarction (Morel et al., 2009), and elevated levels of TF-bearing EVs have been associated with a higher risk of venous thromboembolism (Date et al., 2017) in cancer patients, and mortality in HIV (Mayne et al., 2012), Influenza A/H1N1 (Rondina et al., 2016), and severe COVID-19 infection (Guervilly et al., 2021; Rosell et al., 2021) where the plasma of patients is capable of inducing an increase in TF mRNA levels in neutrophils of healthy donors, promoting the release of TF enriched extracellular traps (NETs) (Skendros et al., 2020). In addition, NETs are associated with microthrombi formation and with deposited platelets in the lungs of COVID-19 patients, which are associated with disease severity (Middleton et al., 2020).

To get an overview of the proteome of EVs in COVID-19, we subjected the dataset to different comparative analyses. Initially, we applied a cluster analysis to all proteins identified in Control *vs* Survivor *vs* Non-survivor, and a few biological processes resulting from these enriched terms stood out: “complement and coagulation cascades,” “platelet degranulation,” and “formation of fibrin clot.” Next, the disease groups (S and NS) were merged, constituting the COVID-19 group to identify from the set of all identified proteins, and only DEPs and EPs were compared with controls. Differential analyses were also performed to verify DEPs and EPs between survivors and non-survivors.

Increasing evidence suggests the involvement of the complement system in the physiopathology of COVID-19 associated coagulopathy. Increased deposition of C3d, C4d, and C5b-9 (membrane attack complex, MAC) was found in the lungs of critically ill COVID-19 patients who suffered a generalized thrombotic microvascular injury (Magro et al., 2020). Plasma levels of C5b-9 were higher in patients requiring hospitalization and invasive mechanical ventilation compared to those discharged from emergency rooms (Ma et al., 2021). Although activation of complement and coagulation cascades in severe COVID-19 has been reported previously (Perico et al., 2021; Overmyer et al., 2021; Afzali et al., 2022), including modulated by the expression of TF (Eslamifar et al., 2020), our findings indicate that few complement proteins are regulated in both survivors and non-survivors of COVID-19. Supported by previous studies, we suggest that complement factors should be consumed after overactivation. Indeed, Sinkovits et al. (2021) noted an increase in markers of complement activation in different COVID-19 severity groups that were dramatically reduced in non-survivors, indicating overactivation, consumption, and dysregulation of the complement system. In summary, the consumption of some of these factors has a strong association with the severity of COVID-19, which supports the investigation of complement inhibitors with a potential effect on mortality in critical patients (Vlaar et al., 2020; Mastaglio et al., 2020; Lim et al., 2021).

In our proteomic analysis, C4b (C4b-binding protein beta chain) and coagulation factor VIII were upregulated in COVID-19 patients. C4b actively participates in the control of the classical pathway of complement activation and interacts with anticoagulant proteins S and serum amyloid P component, which comprise prominent biological processes such as blood coagulation, complement activation, and innate immune response. Moreover, proteins related to the inflammatory response and tissue injury, including proteins S100-A8, S100-A9, serum amyloid A-1, serum amyloid A-2, and immunoglobulins, were significantly increased in the COVID-19 groups, resulting from SARS-CoV-2 infection. Consistent with these findings, Mao and colleagues applied EV proteomics with the intention of monitoring recovered COVID-19 patients without adjacent disease and evaluating the biological status of these individuals after discharge. In their work, differential proteins related to coagulation and inflammatory reactions are described, which suggests attention to the risk of reinfection, hemorrhage, and organ dysfunction even months after hospital discharge (Mao et al., 2021).

Platelet degranulation was strongly enriched with proteins identified in S and NS (cluster 3, **Figure 6**). In this respect, our datasets, and others (Hottz et al., 2020; Manne et al., 2020; Rampotas and Pavord, 2021), attribute to platelets several cellular and molecular mechanisms that contribute to the hypercoagulable state of COVID-19. During the acute SARS-CoV-2 infection, platelets circulate with increased surface expression of activation markers (Zhang et al., 2020) and are also hyperresponsive to stimulation with agonists, leading to higher activation and aggregation compared to platelets of healthy individuals (Manne et al., 2020). Increased formation of platelet aggregates (Rampotas and Pavord, 2021) and platelet–leukocyte aggregates (PLAs) (Manne et al., 2020; Joncour et al., 2020; Hottz et al., 2020; Hottz et al., 2022; Hottz and Bozza, 2022) is also associated with severe COVID-19. Patients present increased proportions of both platelet–monocyte and platelet–neutrophil aggregates, correlating with elevated levels of IL-6, C-reactive protein and severity (Joncour et al., 2020). In line with these data, according to the differential significance indices, COVID-19-specific adhesive proteins were quantified (von Willebrand factor, fibrinogen, thrombospondin-1). These proteins with soluble activators are secreted by platelets during the formation of thrombi that trigger the thromboinflammatory process so characteristic of severe COVID-19.

Reporting proteomic changes among patients with different severity statuses or clinical outcomes is an interesting way to provide relevant clinical information on the progression of COVID-19. Previous studies were instrumental in understanding the molecular mechanisms associated with critical/non-critical infection (Barberis et al., 2021), patients with mild, moderate, and severe infection (Su et al., 2020), and even post-recovery individuals (Mao et al., 2021) compared to healthy donors.

Regarding the discriminative proteome between survivors and non-survivors, we observed a similar protein profile in both analysis groups, as demonstrated in the PCA (**Figure 5B**), in which the groups remained close to each other. Furthermore, the differential abundance profile signaled 18 DEPs (**Figures 7D,F**), and the distribution of cluster patterns indicates similar patterns between survivors and non-survivors in most of the formed clusters (**Figure 6A**). Interestingly, distinctions among proteins considered exclusive to each condition could be observed, 82 being attributed to survivor and 117 to the non-survivor analysis group. The severe clinical condition of the patients, the majority of whom (all NS patients and 4 of the 5 S patients) underwent mechanical ventilation, is a factor that may contribute to the difficulty in distinguishing the two groups by a specific molecular profile.

Thus, we evaluated the proteins punctually and observed findings as a tissue factor pathway inhibitor (TFIP) exclusively in the proteome of non-survivors. According to White and colleagues, TFIP was found to be elevated in a cohort of critically ill patients compared with non-critical patients with COVID-19 (White et al., 2021). With other indicators, the authors hypothesized that in patients with COVID-19, thrombosis could be associated with a localized effect, including on the endothelial surface, and does not necessarily reflect global hypercoagulability of plasma. Interestingly, CD44, highlighted as a centrality protein among the network built with the EV proteome of non-survivor, was also identified, with other important proteins referring to extravasation cells, was associated with severity in a comparative study between patients with moderate and severe COVID-19 (Chua et al., 2020). We also observed thrombospondin (ID: P35442) and basement membrane-specific heparan sulfate proteoglycan core protein (ID: P98160) in the non-survivor proteome, both associated with cell adhesion mechanisms. Additionally, inflammatory activating proteins were also found in the non-survivor proteome, such as olfactomedin-4; myeloperoxidase and heat shock proteins; and a small group of enzymes such as fructose-1,6-bisphosphatase 1; fructose-bisphosphate aldolase and glucose-6-phosphate isomerase, part of energetic metabolism, are also found in the EV proteome of the NS group.

Among the EV proteome of the survivor group, the GP-Ib beta chain (P13224), CXCL7 (P02775), and the platelet-activating factor acetylhydrolase (Q13093) were found to be exclusive. Interestingly, superoxide dismutase 2 (P04179) is present in the *S* group proteome, which can indicate a protective role of EVs among those patients. Additionally, heparanase, the only known heparan sulfate-degrading enzyme, is related to the *survivor* proteome, while the component core protein of the basement membranes Perlecan (P98160) is associated with the *non-survivor* proteome. This versatile proteoglycan is involved in a vast cellular process including cell adhesion, inflammation and autophagy, for example (Gubbiotti et al., 2017), and perlecan/LG3 fragment has also been identified among apoptotic exosome-like, a component of the secretome of apoptotic cells, compared with apoptotic bodies properly (Tucher et al., 2018). Another representative group of proteins on the S proteome is associated with cytoskeleton organization, such as cofilin-1 (P23528); vinculin (P18206); small G protein Rac2 (P15153); ras-related protein Rab-1A (P62820); can also be associated with platelet reorganization during the activation process, due to the majority source of circulating EVs.

Overall, our findings show that circulating EVs exhibit a procoagulant profile and reflect profound proteomic changes in patients with severe COVID-19. The proteomic and functional analysis applied to EVs identified proteins mainly related to activation of the coagulation and complement cascades and platelet degranulation, reinforcing data from our colleagues and indicating that extracellular vesicles are impacted by SARS-CoV-2 infection. We also assigned a set of proteins to the survivor and non-survivor groups, which could be likely markers of severe COVID-19.

## Data Availability Statement

The raw data of mass spectrometry for this study can be found in the PRIDE (https://www.ebi.ac.uk/pride/archive/) under code PXD032808 and other datasets supporting the conclusions of this article will be made available by the authors to any qualified researcher.

## Ethics Statement

The studies involving human participants were reviewed and approved by the National Review Board of Brazil (Comissão Nacional de Ética em Pesquisa - CONEP, CAAE: 30650420.4.1001.0008). The patients/participants provided their written informed consent to participate in this study.

## Author Contributions

EM organized, collected, analyzed, interpreted the proteomic data, and wrote the manuscript. RM-G analyzed, interpreted the data of flow cytometry, and wrote the manuscript. LS interpreted the proteomics data, performed SEC isolations, functional analysis, and wrote the manuscript. RM and SM performed and analyzed the statistical analysis. IA-Q collected and organized clinical samples. FB and TS enrolled patients in the study and conducted clinical surveillance. MT performed MS experiments. MT and PB performed the study designed and supervised the project. EH, JP, HC-F-N, PB, and MT engaged in the conception of the study and contributed to the review and editing of the manuscript. All authors contributed to the article and approved the submitted version.

## Funding

This work was supported by the Conselho Nacional de Desenvolvimento Científico e Tecnológico (CNPq) Network for studying COVID-19 pathogenesis (Grant # 401700/2020-8), the Fundação de Amparo à Pesquisa do Estado do Rio de Janeiro (FAPERJ) (Grant # E26/200.992/2021), the Inova Covid-19 Program (Grant # VPPCB-005- FIO-18-2-74), the Coordenação de Aperfeiçoamento de Pessoal de Nível Superior (CAPES, Grant # 88887.506989/2020-00), and the National Institutes of Science and Technology Program (INCT) on Diseases of Neglected populations (INCT-IDPN, Grant # 465313/2014-0).

## Conflict of Interest

The authors declare that the research was conducted in the absence of any commercial or financial relationships that could be construed as a potential conflict of interest.

## Publisher’s Note

All claims expressed in this article are solely those of the authors and do not necessarily represent those of their affiliated organizations, or those of the publisher, the editors and the reviewers. Any product that may be evaluated in this article, or claim that may be made by its manufacturer, is not guaranteed or endorsed by the publisher.
